# Women with disabilities’ experiences with respectful maternity care in Nepal: a phenomenological study

**DOI:** 10.1186/s12884-025-08398-y

**Published:** 2025-11-25

**Authors:** Savo Noori, Francesca Lanzarotti, Laura Herren, Hridaya Raj Devkota, Kate Roll, Sara Hillman

**Affiliations:** 1 UCL EGA Institute for Women’s Health, Medical School Building, 74 Huntley Street, London, WC1E 6AU UK; 2 UCL Institute for Innovation and Public Purpose, 11 Montague St, London, WC1B 5BP UK; 3 UCL, Epidemiology and Public Health, Gower St, London, WC1E 6BT UK

**Keywords:** Healthcare discrimination, Nepal, Phenomenological study, Respectful maternity care, Women with disabilities

## Abstract

**Background:**

Respectful maternity care (RMC) is essential to safe, dignified childbirth, yet women with disabilities in Nepal face unique barriers in accessing such care. Limited evidence exists on their experiences in relation to the White Ribbon Alliance’s RMC Charter.

**Objective:**

To explore the lived experiences of women with disabilities regarding respectful maternity care in the semi-urban outskirts of Kathmandu, Nepal, and to identify priorities for improving maternity care.

**Methods:**

A phenomenological study design was employed between April and May 2023. Data collection included 12 in-depth interviews with women with disabilities, 7 with healthcare providers, and 2 focus group discussions with 11 Female Community Health Volunteers. Interviews were audio-recorded, transcribed, translated into English, and thematically analyzed, guided by the RMC Charter.

**Results:**

Women’s experiences varied widely. Negative accounts included disrespect, poor communication, compromised privacy, and structural barriers such as overcrowded facilities and lack of disability-friendly infrastructure. Economic constraints and transport challenges further limited timely access to care. Some providers perceived women with disabilities as having limited autonomy, though most women reported making their own healthcare decisions. Positive experiences included respectful communication, practical support, and personal assistance from providers.

**Conclusion:**

Women with disabilities in the Kathmandu outskirts encounter disability-specific barriers to respectful maternity care, including provider attitudes, inadequate communication, and inaccessible facilities. We recommend integrating disability-focused RMC training into health professional curricula, investing in disability-friendly infrastructure, and adopting policies that safeguard privacy, dignity, and autonomy.

## Background

Worldwide, the field of maternal and neonatal health has seen remarkable improvements, resulting in reduced rates of maternal and neonatal deaths [1]. However, disparities in healthcare access and outcomes persist, particularly pronounced among low-to-middle income countries and within various socioeconomic strata [2, 3]. A notably vulnerable group is women with disabilities, who confront multifaceted challenges in accessing essential maternal healthcare services [4, 5]. These challenges are often compounded by social, ethnic, and cultural barriers, alongside an unwelcoming reception at healthcare facilities, lack of privacy, information, and respect [6, 7].

The issue of respectful maternity care (RMC) is central to this discourse. The World Health Organization (WHO) defines RMC as “the care organized for and provided to all women in a manner that maintains their dignity, privacy and confidentiality, ensures freedom from harm and mistreatment, and enables informed choice and continuous support during labour and childbirth [8].” Inadequate RMC is a major barrier for women considering facility-based deliveries [6, 9–11]. The absence of such care not only diminishes patient satisfaction but also mediates accessibility to skilled maternity care, influencing the likelihood of returning for future deliveries [12, 13].

The White Ribbon Alliance’s RMC Charter, published more than a decade ago, encapsulates the fundamental rights of childbearing women, highlighting the necessity of freedom from harm, informed consent, privacy, dignity, equality, timely healthcare, and autonomy [14]. The WHO’s emphasis on dignity and respectful care during pregnancy and childbirth underlines its global importance [8].

Studies show that women with disabilities face a higher risk of negative maternal health outcomes, including unplanned pregnancies and elevated maternal mortality rates [15, 16]. These disparities are often exacerbated for women with disabilities due to decreased healthcare access [5]. There is evidence that women of lower socioeconomic backgrounds often face higher risks of mistreatment in maternity care [17], but there are few studies examining the women with disabilities’ experience through the principles of RMC [18, 19].

While recent studies by researchers like Devkota et. al. [20] and Morrison et. al. [21] have delved into the experiences of women with disabilities in Nepal, a specific examination of their experiences in the context of RMC, particularly concerning the principles outlined by the White Ribbon Alliance, has not been conducted. Given this context, this study aims to contextualize the rights of childbearing women with disabilities in Nepal within the framework of the RMC Charter. By doing so, it seeks to contribute to the improvement of maternal healthcare for this marginalized group.

## Methods

### Study design

This study employed a phenomenological design to explore the lived experiences of women with disabilities in maternity care. Data were generated through semi-structured interviews (SSIs) and focus group discussions (FGDs) as part of a broader collaboration between the Reach Alliance and University College London (UCL). The present paper draws on a subset of this larger study, focusing specifically on respectful maternity care.

### Ethics

The study was approved by the UCL Research Ethics Committee and the Nepal Health Research Council.

### Study setting

This study was conducted on the outskirts of Kathmandu, Nepal, specifically in the areas of Nagarjun, Dakshinkali, Kirtipur, Bhaktapur, Ramkot, and Basantapur. These sites fall within the Kathmandu district of Bagmati Province, as defined in 2011 by the Government of Nepal [22]. According to the Central Bureau of Statistics (2022), the Kathmandu district has a disability rate of 1.4% (1.3% among females and 1.5% among males) within a population of 2,041,587 [23]. While the urban centre of Kathmandu is rapidly urbanising, the outskirts present a blend of traditional and modern lifestyles [24, 25].

The outskirts of Kathmandu were selected as they represent a mix of urban and rural settings, capturing diverse socioeconomic and cultural perspectives. Women with disabilities in these areas often face additional barriers, such as transport and accessibility challenges, compared with those in central urban facilities [20]. Furthermore, these sites were recommended by local collaborators to ensure both feasibility and diversity in participant recruitment within the study timeframe.

### Participant recruitment and characteristics

The final sample included 12 women with disabilities, 7 healthcare providers, and 2 FGDs with a total of 11 FCHVs. Sample size was guided by the principle of thematic saturation, reached when no new themes emerged in later interviews and focus group discussions.

The recruitment strategy included opportunity sampling through contacts in local communities, volunteer sampling during the field trip, and snowball sampling within participant networks. Women with disabilities were eligible if they were aged 18 or older, had been pregnant within the last five years but were not pregnant at the time of interview, and fell under one of Nepal’s ten recognized disability categories (physical, vision-related, hearing-related, vocal and speech-related, deaf-blind, intellectual, mental and psychosocial, haemophilia, autism, and multiple disabilities) [25]. Women with mental disabilities who could not provide informed consent independently were excluded.

Healthcare professionals were recruited purposively through local health facilities to ensure representation from different levels of the healthcare system, including medical doctors, senior auxiliary nurses, and staff nurses. Initial contacts were facilitated by local collaborators, and additional participants were identified through snowball sampling. The FCHVs were recruited from Dakshinkali and Nagarjun municipalities with support from local coordinators.

### Data collection and analysis

Data were collected over a five-week period (April–May 2023) through SSIs with women with disabilities and healthcare providers, and two FGDs with FCHVs. Three different topic guides were created for each participant group based on a thorough literature review. To ensure contextual appropriateness, the topic guides were reviewed by local researchers based in Nepal, including a senior researcher with expertise in disability and maternal health, together with two research assistants experienced in research on women with disabilities. Their feedback helped refine the language and ensure cultural relevance.

Interviews were conducted in private settings for participant comfort and privacy. Except for four healthcare providers who spoke English, the two Nepali female research assistants conducted interviews with the women and discussions in their native language. This approach aimed to mitigate potential power imbalances and ensure authenticity of the data collected. Prior to each interview, participants were given an information sheet that detailed the objectives of the project. Additionally, informed consent was obtained through a consent form, which outlined confidentiality, anonymity, and the option to withdraw. The data was audio-recorded with participant’s permission.

Post-data collection, interviews were transcribed verbatim and then translated to English. To ensure privacy, identification numbers and alphabetical characters were used, and personal identifiers were removed from the data. The team conducted a double-coding procedure to ensure the reliability of the data, which was then subjected to thematic analysis for extracting key themes and insights. Thematic saturation was reached after the second FGD, as no new themes emerged. In line with qualitative research standards, sample adequacy was guided by the principle of saturation rather than a predetermined minimum number of FGDs or participants [26].

## Findings

### Demographics

The study included 12 women with disabilities, 7 healthcare providers, and 11 FCHVs. The women ranged in age from 21 to 42 years and represented diverse socio-economic and caste backgrounds. Most had experienced their first or second pregnancy within the last five years; none were pregnant at the time of interview. For those with more than one birth, interviews emphasized their most recent pregnancy and delivery.

Ten of the women had completed some level of formal schooling within Nepal’s national education system, ranging from primary to higher education, while two had never attended school. Disabilities included visual impairments (low vision to complete blindness) and physical impairments (partial mobility limitations to severe disability requiring assistive devices), with severity categorized according to Nepal’s official classification system.

The healthcare provider group comprised three medical doctors (including one with 25 years’ maternal health experience), two senior auxiliary nurses, and two staff nurses. The 11 FCHVs, drawn from Dakshinkali and Nagarjun municipalities, had extensive experience in community-based maternal care.

Although the study was guided by the RMC Charter, not all domains were represented in participants’ accounts. Consistent with the phenomenological approach, only the principles that emerged directly from the data are reported here. Thematic analysis revealed six overarching themes: (1) freedom from harm, dignity, and respect; (2) information, consent, and respect for choices; (3) privacy and confidentiality; (4) equality, non-discrimination, and equitable care; (5) timely healthcare and the highest attainable health; and (6) liberty and autonomy.

### Right to be free from harm/ill-treatment and the right to dignity and respect

Variations were observed in the treatment received by women from healthcare providers during their visits. For about half of these women, their right to be free from mistreatment, along with their right to dignity and respect, was not upheld. Some participants reported negative encounters that left them feeling devalued and mistreated within the healthcare system. These experiences were particularly pronounced in government hospitals where the quality of interpersonal interactions often fell short of respectful care standards. One participant’s narrative highlighted the severity of such encounters: *“The health workers used to speak bad words. I felt disappointed at that time. After that*,* I decided not to visit a governmental hospital again for a health checkup so I went to a private hospital during my second pregnancy”* (W9).

Several women described mistreatment that was specifically linked to their disability, including being asked intrusive questions about its cause or being treated as less deserving of attention compared to women without disabilities. One participant explained: *“They used to ask me ‘how did this happen to you?’ and ‘from what age did this happen?’ They used not to pay attention to my health issues and did not treat me like people without disabilities”* (W7). During childbirth, some women reported behaviours such as *“scolding”* (W12), which further undermined their sense of dignity and respect.

Healthcare providers themselves acknowledged shortcomings in interpersonal care. One explained: *“In a place like a teaching hospital as everyone is knowledgeable but in behaviour*,* there is nothing there”* (H7). A further provider reflected on systemic pressures: *“Knowledge is there*,* but when there are too many patients*,* we cannot give time for respect”* (H6). Such admissions reinforced women’s accounts but also revealed a divergence in perspective—what women experienced as mistreatment, providers explained as system-driven constraints.

Conversely, about half of the women had favorable interactions with their healthcare providers: *“Everything was good for me. They treat me in a very good manner”* (W3). For instance, two women appreciated the extra support from their healthcare providers who gave them personal contact numbers for emergency use, with one stating: *“They also gave me a phone number and said that we would help you whenever and wherever needed”* (W9). All the women agreed that positive attitudes and respectful care were instrumental in fostering trust and a sense of security in their healthcare experiences.

### Right to information, informed consent, and refusal, and respect for choices and preferences

The women’s accounts highlighted a varied landscape of communication, information exchange, and consent practices within the maternal healthcare system during their pregnancies.

Responses were divided into two clear groups. Less than half of women shared favorable experiences, highlighting that healthcare providers were attentive, welcoming of questions, and provided thorough information. One woman expressed her satisfaction: “I got enough information here that I did not want to go anywhere else” (W9). Members of this group appreciated the sufficient time allocated, the detailed explanations regarding pregnancy risks, and the clarity and effectiveness of communication. This method resulted in their overall contentment, as they felt all their questions were fully answered.

However, for slightly more than half of the women, communication was often poor. Information was rarely offered voluntarily, and the women found themselves having to actively solicit it: “But they did not counsel themselves. They only said whenever we asked them” (W10). Remarks such as “they did not counsel very well” (W2) led to feelings of being unsupported and uninformed about their health conditions during pregnancy among the women. Additional worries were raised regarding the understanding of diagnostic results, like blood tests and ultrasound scans. A number of women believed that the descriptions given by healthcare providers were inadequate, frequently condensed into the vague statement, “it’s okay” (W4). This ambiguity caused uncertainty about their health condition. The same woman expanded on this point: “What does okay mean? Not everyone is a medical person. It is not possible. Not everyone is educated.” (W4).

The majority of healthcare providers acknowledged challenges in communication during consultations with women with disabilities: “Communicating with them, that’s definitely a challenge” (H4). Despite this, most were willing to dedicate extra time for thorough explanations: “We will try to counsel them until they understand well, until they are satisfied with our services… I think giving 5–10 minutes more will not spoil anything” (H5). However, this intention often clashed with the reality of a demanding patient flow: “We have a very heavy patient workload, and due to worker stress, many of the healthcare providers couldn’t give them quality time” (H3).

While communication was often a challenge, only one healthcare provider mentioned the utilisation of alternative communication methods, such as sign language and written materials. Similarly, only one participant from the FCHVs mentioned such methods: “Showing pictures, flex, and flipcharts makes understanding easier. We use this flipchart for nutrition counseling, and it is more effective than speaking” (FCHV Ramkot), indicating while these methods of relaying information were useful, they were not used frequently.

### Right to privacy and confidentiality

The concerns of women regarding privacy and confidentiality were primarily linked to overcrowding and the handling of sensitive information. Severe overcrowding in facilities compromised their privacy, as one woman described: *“Two pregnant mothers used to stay in the same bed. I barely found a space”* (W10). Such conditions denied women the private space they needed during pregnancy and childbirth, directly violating their right to confidential care.

In addition to concerns about physical privacy, women and providers highlighted challenges to confidentiality in the handling of sensitive information. Healthcare providers frequently communicated private information through the patients’ relatives rather than directly to the women themselves, often without obtaining explicit consent. This approach, especially for women with hearing difficulties, was noted by a healthcare provider: “If they have hearing problems then we encourage them to bring their visitors” (H3). Additionally, over-reliance on family members for communication was particularly problematic during critical times like delivery, when family presence was typically restricted: “Inside the labour room, family and friends are not allowed to come inside” (H4). Both women and providers highlighted the central role of relatives in communication, though what women experienced as a breach of confidentiality was often seen by providers as a practical necessity.

### Right to equality, freedom from discrimination, and equitable care

Most healthcare providers indicated that they do not discriminate against women with disabilities. Yet, in practice, some attitudes within the healthcare system contrasted with these claims. For example, a common misconception about the physical strength of women with disabilities led to biased medical advice: “Doctors also think that she can’t do normal delivery, she has to go through a caesarean. I have faced this as during my first delivery” (W4).

Accounts also pointed to both equitable practices and discriminatory assumptions within maternity care. On the positive side, several women reported priority access at facilities, such as “People with disabilities don’t have to stand in line” (W9). Providers confirmed this accommodation: “We prioritize them while providing services” (H6), reflecting efforts toward equitable treatment. At the same time, disability-related stereotypes continued to shape care. Providers often framed their approach as formally equal, stating, “The disability doesn’t count because she is a woman with a pregnancy… That’s how I would certainly treat her” (H2). Others cautioned against segregating women with disabilities, noting, “Putting them in a different basket… creates a lot of difference… everything should be together, but services… oriented to the needs of each individual” (H4). Taken together, these accounts reveal a perceptual gap: providers articulate equal intent, while women describe bias in assumptions that can influence decision-making and undermine equitable care.

### Right to timely healthcare and to the highest attainable level of health

The women reported significant obstacles in accessing healthcare in a timely manner, primarily due to the distance to facilities and transportation challenges. One participant described her journey to the health center as, “It’s long, one hour long” (W5), a sentiment echoed by a healthcare provider who mentioned: “It’s difficult to reach healthcare facilities, and public transportation facilities are not available all the time” (H2).

Women with disabilities consistently reported challenges posed by the physical infrastructure of healthcare facilities. A predominant sentiment conveyed by the women was that these facilities were not designed with their specific needs in mind: “When I used to visit the healthcare facility, I found it not to be disabled friendly” (W7). A significant concern was the presence of stairs without alternative provisions such as ramps or lifts, making navigating through the facilities challenging. One woman elaborated: “It was difficult for us to climb the stairs. There were staircases. It would have been easier if there had been a ramp and a lift” (W4). Notably, a rare exception to this experience was mentioned by another woman who found relief in one facility that provided a “lift” (W6); however, it is worth noting her care was at a private facility. Furthermore, specific concerns like “slippery floors” (W6) added to the apprehension of potential risks while navigating these spaces.

Financial barriers also played a critical role in limiting timely care. Providers and FCHVs consistently identified cost as a major barrier: “I think the first one [barrier] is the financial barrier” (H2); “If the economic status is low then everything has become low. So, the economic status of a family matters everything” (H5). FCHVs also observed that wealthier women tended to bypass government facilities: “The higher-class people even don’t come to this facility [governmental health post], they used to visit tertiary level private hospitals directly” (FCHV Dakshinkali). Some private hospitals offered discounts for women with disabilities, leading to facility switching: “The initial hospital didn’t provide discounts for disabled individuals, so I switched to another” (W3).

Beyond timeliness, women also described quality-related concerns that affected their experiences of care. Some voiced discomfort due to frequent medical doctor rotations: “Today used to be one doctor, tomorrow used to be another doctor. If I consulted with the same doctor it would have been better; however, it was not like that” (W7). Additionally, several women experienced delays in seeing their newborns due to procedural or administrative reasons in healthcare facilities. These delays had a significant emotional impact, as one woman expressed her distress and confusion during such a moment: “I couldn’t see my baby so I asked the doctor where is my baby?… my husband thought that I lost my child” (W4). The waiting periods extended their effects beyond the women to their families, who faced anxiety and uncertainty while awaiting news.

### Right to liberty and autonomy

The decision-making process in healthcare for women with disabilities in Nepal presented a complex picture, with providers often perceiving decisions as family-driven while women described more autonomous experiences.

The healthcare providers and the FCHVs generally viewed these women as having limited involvement in their healthcare decisions, often influenced by family members: “They’re not involved much in their treatment process. They seek advice from their parents, husbands, and first-degree relatives and then they ask us for the best options and which to do accordingly” (H3). The level of autonomy was also seen as dependent on the type and severity of the disability: “If women experience solely a physical disability, they can independently make decisions” (H5).

Contrasting with these observations, the women themselves reported diverse experiences in decision-making. Some exercised complete autonomy – “This is my own decision [to get pregnant] (W5)” – while others involved their families in the process: “We discussed it together in the family and made the decision” (W10).

In healthcare settings, women’s experiences varied from being guided by medical advice to actively asserting their choices. For example, one woman recounted aligning with a doctor’s recommendation: “And about the place of delivery, the doctor suggested we deliver at the hospital. So, we made a plan to deliver our baby to the hospital” (W1).

Furthermore, women’s autonomy in choosing between caesarean sections and natural deliveries also varied. While some women reported that c-sections were recommended to them due to their disability, the majority felt their decisions were respected: “When I asked if there can be a normal delivery they said that normal delivery was possible in case of breech cases too and they also warned that there were risks. It was like a warning. But the decision was my own” (W5).

## Discussion

Our findings highlight provider attitudes, systemic barriers, communication, and autonomy as the most significant variables influencing respectful maternity care for women with disabilities. These align with global studies [5, 7, 20].

Findings from this study provided a range of perspectives on respectful maternal care, drawing insights from both women with disabilities and healthcare providers, including FCHVs. In terms of respectful attitudes from healthcare providers, the experiences of women were notably divided. While a considerable number of women reported receiving respectful care, a similarly substantial number faced significant challenges, especially in government hospitals. In these settings, more frequent reports emerged of compromised rights, devaluation, and mistreatment. This variation in experiences is consistent with findings from other studies conducted in Nepal. For instance, a recent study identified a significant degree of mistreatment in the context of maternity care [27]. Interestingly, the varied experiences documented in this study contrast with findings from Gurung et al. [28], who conducted a study in Nepal that generally showed a favourable experience of women with RMC. However, the study did not include women with disabilities. Perhaps the difference in findings could be attributed to the specific challenges faced by women with disabilities, who may encounter unique barriers and biases in the healthcare system. Interestingly, Gurung et.al’s [28] study also showed that people of lower socioeconomic status received lower quality care and data from Nepal show that those with disabilities have lower socioeconomic status [29].

Interestingly, the varied experiences documented in this study contrast with findings from Gurung et al. [28], who conducted a study in Nepal that generally showed a favourable experience of women with RMC. However, their study did not include women with disabilities. The difference in findings may therefore reflect the unique challenges faced by women with disabilities, who encounter barriers such as stigmatizing assumptions about their ability to give birth, inaccessible infrastructure, and reliance on relatives for communication. These disability-specific issues could help explain why Gurung et al.’s participants, largely women without disabilities, reported more positive experiences. Gurung et al. also showed that women of lower socioeconomic status received lower quality care, and data from Nepal indicate that people with disabilities are disproportionately represented in lower socioeconomic groups [29]. Together, these factors suggest that disability and socioeconomic disadvantage intersect to shape more negative maternity care experiences.

Furthermore, systemic barriers significantly hindered the provision of RMC. These included infrastructure challenges such as distance, transportation, and the lack of disability-friendly facilities within healthcare centres, impacting timely access to care. Similar systemic barriers have been observed in other studies [5, 20, 30].

Women with disabilities in our study linked public hospitals to crowding, poor privacy, brusque communication and weak continuity, and private facilities to calmer, more respectful, personalized care. This mirrors LMIC and Nepal evidence: private sites often score higher on responsiveness, while public services face caseload and space constraints that undermine dignity [31–34]. In Nepal specifically, comparative work has documented quality gaps across facility types and suggests technical/perinatal care indicators are often better in private facilities, while public sites struggle with crowding and continuity—patterns that help explain the interpersonal deficits described by our participants [32]. Importantly for disability, these sector differences shape *how* RMC is (or isn’t) realized: women in public settings described privacy breaches (e.g., being overheard, information relayed through relatives) and disrespect that heightened stigma, whereas private settings sometimes mitigated these harms by offering calmer environments and more individualised communication.

Regarding communication, the methods employed by providers varied based on the specific nature of the disability. Providers often used non-direct forms of communication, frequently through the women’s companions, particularly when dealing with women with hearing or speech disabilities. The use of alternative communication techniques, such as sign language or visual tools, was infrequently employed. Research indicates that poor communication can result in various adverse effects, such as reduced patient autonomy and heightened maternal anxiety, emphasizing that women place high importance on effective communication [30, 35, 36]. An additional repercussion arising from communication gap is the potential impact on informed decision-making. RMC is centered on providing all women, including those with disabilities, with comprehensive information and choices [14]. However, the communication barriers highlighted might limit the ability of these women to make fully informed decisions about their maternity care. This issue, as supported by findings in other studies [19, 37, 38], emphasizes the critical need to address these communication challenges to ensure that principles of respectful maternity care are met, allowing all women access to the necessary information for making well-informed healthcare decisions.

Moreover, involving family members in healthcare communication while often adopted out of necessity, it can inadvertently compromise the privacy and breaches of confidentiality, which are crucial elements of RMC [14].

Finally, this study revealed a clear disparity in perceptions of autonomy. Women with disabilities often reported making their own decisions or collaborating with their spouses on pregnancy and healthcare choices. In contrast, the healthcare providers and the FCHVs tended to view these women’s decisions as heavily influenced by their families. The contrast in perceptions between the women and their healthcare providers highlights a critical gap in understanding and respecting patient autonomy and might be due to insufficient training in understanding disability rights and capabilities. The implications of this perceived lack of autonomy are significant. When women with disabilities are unjustly seen as dependent and incapable of decision-making, healthcare providers may unintentionally marginalize them in medical discussions, failing to involve them directly. This exclusion can lead to distress, result in care that does not adequately cater to their specific needs, and open the possibility of miscommunication and misinformation.

To significantly enhance the principles of RMC for women with disabilities in Nepal, focused interventions are necessary. Central to these interventions is the comprehensive training of healthcare providers. Such programs could concentrate on improving communication skills and fostering the core principles of RMC, such as dignity, privacy, and respect in patient interactions. This training would equip healthcare providers with the skills and sensitivity required to deliver care that adheres to RMC standards, ensuring that women with disabilities receive not just access to healthcare but also respectful and dignified treatment. Research exploring the impact of such training in Ethiopia and Ghana reveals promising outcomes [39, 40]. Additionally, infrastructure development should align with RMC principles and create healthcare settings that not only accommodate physical access but also foster an environment where respect, confidentiality, and patient-centred care are the norm [8]. Facilities should be designed and equipped to support the unique needs of women with disabilities in a manner that upholds their dignity and autonomy. Research conducted by Asefa et al. [41] indicates that while training is essential, it alone is insufficient for effective respectful maternity care implementation without simultaneously addressing other factors like infrastructure.

### Limitations of study and further research

The authors acknowledge several limitations of the study. Due to time constraints on fieldwork, the study had a relatively small sample size. While the aim was to include a diverse range of disabilities, the majority of participants were women with physical and visual disabilities. Additionally, the participants were primarily from the outskirts of Kathmandu, indicating that the perspectives represented are specific to that region. Despite careful efforts to minimize power imbalances between researchers and participants, the cross-cultural aspect of the research may have inadvertently allowed some imbalances to remain. This was mitigated by conducting interviews with the women through a local team in their native language.

Considering Nepal’s varied landscape and cultural diversity, future research could usefully compare maternal healthcare communication challenges across urban and rural settings. Expanding the scope to include a broader range of disabilities may also reveal challenges unique to each group. Building on our findings, future studies should adopt mixed-methods and longitudinal designs. Quantitative research could measure the prevalence of respectful and disrespectful maternity care experiences among women with disabilities, while qualitative approaches could further illuminate cultural and contextual dynamics. Participatory and community-based research would additionally empower women with disabilities to co-produce knowledge, ensuring that interventions remain responsive to their priorities.

## Conclusion

This study aimed to explore the experiences of women with disabilities in Nepal regarding RMC principles. Women’s experiences with healthcare provider attitudes varied, ranging from positive to unsatisfactory. Disability-specific challenges in communication were prevalent, often involving family members and lacking in alternative communication methods. Inadequate healthcare infrastructure and amenities frequently compromised privacy and diminished care quality. Both healthcare providers and FCHVs tended to view women as lacking autonomy. Recommendations include training providers in disability-sensitive RMC practices and restructuring healthcare systems to prioritize patient dignity and privacy.

## Data Availability

The datasets generated and/or analyzed during the current study are available from the corresponding author on reasonable request.

## Abbreviations

FCHVs: Female community health volunteers
FGDs: Focus group discussions
GoN: Government of Nepal
RMC: Respectful maternity care
SSI: Semi-structured interviews
UCL: University college London
WFDB: World federation of the deafblind
WHO: World health organization
